# A Cardioplegic Solution with an Understanding of a Cardiochannelopathy

**DOI:** 10.3390/antiox10121878

**Published:** 2021-11-25

**Authors:** Min Jeong Ji, Jeong Hee Hong

**Affiliations:** 1 Department of Physiology, Lee Gil Ya Cancer and Diabetes Institute, College of Medicine, Gachon University, Incheon 21999, Korea; bwon25@gmail.com; 2 Department of Oral Biology, College of Dentistry, Yonsei University, Seoul 03722, Korea

**Keywords:** cardioplegia, IR injury, ion transport, ion channel, cardiochannelopathy

## Abstract

Cardiac surgeries have been improved by accompanying developing cardioplegia solutions. However, the cardioplegia application presents an ongoing challenge with a view of a sufficiently restored cardiac function. In this review, we focus on the cardioplegia-induced mechanism and summarize the findings of studies undertaken to improve cardioprotective strategies. Currently, and somewhat surprisingly, relatively little is known about cardiac electrolyte regulation through channel physiology. We hope that an improved understanding of the electrolyte transport through ion channels/transporters and modulations of water channel aquaporins will provide an insight into cardiac channel physiology and a channel-based cardiac pathology of a cardiochannelopathy.

## 1. Role of Cardioplegia and Potential Injuries

During cardiac surgery, hyperkalemic cardioplegic solutions are the essential gold standard to prevent cardiac depolarization and temporarily sustain a cardiac arrest through the reduction of cardiac loading. In detail, elevated extracellular K^+^ concentrations (10–40 mM) of hyperkalemic cardioplegic solutions increase the resting myocyte membrane potentials (E_m_) from −85 mV to between −65 and −40 mV, which inactivates fast Na^+^ channels [1]. These higher potentials block the conduction of myocardial action potentials, induce a depolarized arrest, and minimize tissue damage during cardiac surgery. Various cardioplegic solutions based on the component, temperature, or period of delivery have been developed as protective strategies.

Although cardioplegic solutions to protect cardiac cell death from ischemia, detrimental injury may occur during intraoperative ischemia due to multi-dose infusions of a cardioplegic solution or the misdistribution of a solution distal to total coronary occlusions [2]. In addition, there is the potential for a reperfusion injury during each infusion and after the aortic cross-clamp removal [2]. In the case of del Nido cardioplegia, the intracellular pH is maintained and the influx of Ca^2+^ during ischemic arrest is reduced; this has been used to improve myocardial protection [3,4]. However, although del Nido cardioplegia does not lead to clinical side effects, there is an adverse effect. When del Nido cardioplegia was used in an adult aortic root surgery, the ischemic time of the patient was increased compared with blood-based cardioplegia, thereby increasing the myocardial injury Although cardioplegic solutions possess advantages such as successful cardiac surgery to protect an IR injury, diabetic hearts have been shown to have worse clinical outcomes compared with non-diabetic hearts [5,6,7]. Thus, we considered that disease states including diabetes potentially affect a restored cardiac function. In this review, we highlight various potential threat factors of cardiac injuries, describe the related molecular mechanisms in cardioplegia-exposed cardiac tissues for a sufficiently restored cardiac function, and involve several approaches to improve the pathological mechanisms.

## 2. Cardioplegia Solution Characteristics, Risk Factors, and Related Mechanisms

### 2.1. Myocardial Ischemia/Reperfusion Injuries

**Figure 1:**
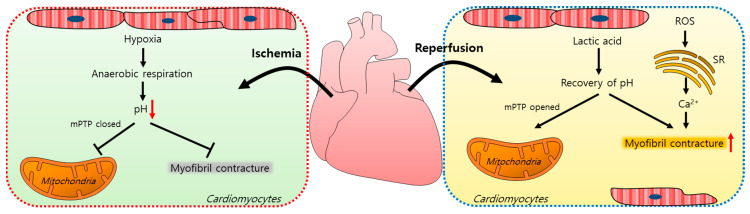
Schematic illustration of the mechanism of an ischemia/reperfusion injury in cardiomyocytes. Ischemia-induced hypoxia results in anaerobic respiration and reduces the intracellular pH, which closes mPTPs and reduces myofibril contracture. During reperfusion, lactic acid accumulates, the intracellular pH increases, and mPTPs open. ROS increase the intracellular Ca^2+^ release from the SR and induce myofibril contracture. mPTP: mitochondrial permeability transition pore; ROS: reactive oxygen species; SR: sarcoplasmic reticulum.

Myocardial ischemia/reperfusion (IR) injuries occur during cardiac surgery and cardioplegic solutions have a protective effect although their effects are limited [8]. In the ischemic state, a lack of oxygen in cells results in ATP and lactic acid production by anaerobic glycolysis and, thus, a reduction in the intracellular pH [9]. Furthermore, the lactic acid generated inhibits the functions of mitochondrial permeability transition pores (mPTPs, a non-selective channel in the mitochondria) and myofibril contracture [10,11]. During a myocardial reperfusion, Ca^2+^ levels increase in the cardiomyocytes due to ROS-induced sarcoplasmic reticulum (SR) dysfunction [11,12], resulting in a reperfusion injury. The pH recovery mediates the opening of mPTPs, which interferes with mitochondrial membrane potentials [10]. Reoxygenation by reperfusion also increases the cytosolic Ca^2+^ levels in cardiomyocytes, causing their hypercontraction and subsequent cell death [12,13,14]. A schematic illustration of the mechanism of an IR injury is represented in Figure 1.

### 2.2. Cardioplegia-Mediated Apoptosis

In this section, we discuss the mechanism of cardioplegia application and associated injuries after a cardioplegic arrest and summarize the numerous studies undertaken to improve the cardioprotective methods against an IR injury over the past several decades. Cold crystalloid or blood cardioplegic solutions have been widely used for cardiac surgery for myocardial protection. However, it has been addressed that cold crystalloid cardioplegia induces early apoptosis signaling events in the myocardial endothelium and cardiomyocytes [15,16]. Two types of cardioplegia methods have been evaluated after a cardioplegic arrest on myocardial injuries in dogs. Both cold blood and cold crystalloid cardioplegia resulted in higher cardiomyocyte apoptosis percentages and an increased caspase-3 expression [17]. To reduce the threat points posed by a cardioplegic arrest, the NHE-1 inhibitor eniporide was applied in a cold cardioplegic or a Krebs–Ringer solution to guinea pigs and was found to improve the relationship of the diastolic left ventricular pressure intracellular Ca^2+^ levels [18]. The cardioplegia group treated with eniporide showed reduced Ca^2+^ loading and infarct sizes [18]. Furthermore, the application of cyclosporine A, a mitochondrial permeability transition pore inhibitor, in the presence of cold crystalloid cardioplegia prevented mitochondrial permeability, the mitochondrial translocation of Bax, and protected the mitochondrial structures in a newborn piglet myocardium [16]. Bax (a member of the Bcl-2 family) triggers the permeabilization of the mitochondria to form pores on the mitochondrial outer membrane; this was increased by cold crystalloid cardioplegia. This translocation of Bax was inhibited by a pretreatment with cyclosporine A. The role of AMP-activated protein kinase (AMPK) on endoplasmic reticulum (ER) stress has been evaluated in a cardioplegic-induced hypoxia/reoxygenation (H/R) injury [19]. AMPK activation dramatically attenuated the H/R-induced apoptosis of cardiomyocytes by increasing the anti-apoptotic (e.g., Grp 78 protein) levels and suppressing the pro-apoptotic signals (e.g., caspase-12 and GADD153 (growth arrest and DNA damage-inducible gene 153)) [19]. The administration of bradykinin (BK) during cold crystalloid cardioplegia decreased oxygen consumption and reduced the myocardial metabolic demands of mild-to-moderate hypothermia [20,21] as well as IR-induced inflammation and apoptosis whereas the inhibition of the BK receptor or nitric oxide synthase diminished the inflammatory and apoptotic responses of rabbit cardiomyocytes [22]. Furthermore, nitric oxide (NO) modulation during a cardioplegic arrest might improve the anti-apoptotic signal profile through the NF-κB/Akt/Bcl-2/Bax axis [19]. More recently, the potential protective effects of the flavonoids astragalin and dihydromyricetin in ischemia during a cold crystalloid cardioplegic arrest were evaluated [23]. A cold crystalloid cardioplegic solution in the presence of astragalin or dihydromyricetin enhanced the anti-inflammatory pathways and attenuated the oxidative signals [23]. In addition to mitochondrial stress, the expression of urocortin (a cardioprotective protein) was investigated under a warm blood cardioplegic arrest [24,25]. More recently, in the heart of a diabetic patient, the downregulation of urocortin was associated with cardiomyocyte apoptosis and the alteration of the nuclear location of PKC-δ [26,27]. Cardioplegia techniques were also compared with the aim of reducing the risk of an IR injury. Retrograde cardioplegia involves the cannulation of the solution into the coronary sinus whereas antegrade cardioplegia involves cannulation into the aortic root [28]. Retrograde cardioplegia causes greater cardiomyocyte apoptosis and caspase-3 activation than antegrade cardioplegia after IR [29]. Although changes in the strategies of cardioplegic solutions have been addressed, there are no clear advantages of one cardioplegic strategy over another. Accordingly, we propose an understanding of the cardioplegia-occurring molecular mechanism with a view of a cellular pathological response.

### 2.3. Cardioplegia-Induced Inflammation and Dysfunction

Cytokine levels play critical roles in inflammatory responses. It is well-known that a cardiopulmonary bypass (CPB) contributes to postoperative complications [30]. The levels of tumor necrosis factor (TNF)-α, interleukin (IL)-6/8, and IL-10 in the blood of patients that had received cold crystalloid or warm blood cardioplegia were compared [30]. They reported that the serum levels of TNF-α, IL-6, and IL-8—excluding IL-10—were higher in the cold crystalloid than in the warm blood-exposed group. Based on their comparative study of cardioplegia trials, it would seem that warm blood cardioplegia may reduce the inflammatory response more effectively than cold crystalloid cardioplegia. Furthermore, an additional supplementation of an amino acid such as L-arginine in cardioplegia reduced the cytokine release; this was significant for IL-6 [31]. In addition to the inflammatory response, hyperkalemic cardioplegia induced cardiomyocyte swelling and reduced contractility in various models [32,33,34]. Treatment with the adenosine triphosphate-sensitive potassium channel (K_ATP_) opener diazoxide also reduced cardiomyocyte swelling and inhibited cardioplegia-mediated reduced contractility [33]. It was revealed that the maintenance of the cardiomyocyte volume was associated with improved contractility and stunning.

### 2.4. Cardioplegia-Induced Oxidative Stress

Oxidative stress induced by CPB mediates myocardium inflammation and damage. To reduce the effect of oxidative stress, a pyruvate-enriched cardioplegia was investigated in pigs [35]. A combination of blood and cold crystalloid cardioplegia with pyruvate increased the tissue inhibitor of metalloproteinase-2 (TIMP-2) contents (a known stimulator of cell migration and apoptosis) and inhibited the metalloproteinase-9 (MMP-9) activity [35]. In addition, the application of pyruvate during cardioplegia reduced CPB-induced myocardial inflammation by increasing the myocardial GSH redox state (GSH/GSSG) and TIMP-2 levels [35]. GSH and GSSG are the mediators of oxidation/reduction reactions and the GSH/GSSG ratio is used as a marker of oxidative stress [36]. Cardioplegia with pyruvate decreased this ratio in the coronary sinus and finally reduced oxidative stress [35]. Moreover, a comparison of inflammatory cytokines after the administration of two types of cardioplegia solution, i.e., a “del Nido” or a “modified St. Thomas” solution, revealed no detectable difference, suggesting that the consideration of the administration intervals is more important than the cardioplegia solution type [37].

Various studies have been performed to improve the arrested status and to abolish the risk factors of cardioplegia such as inflammation, swelling, stunning, and oxidative stress (summarized in Table 1). However, a more effective strategy is still required to properly address these complications. The precise study of the mechanisms responsible for the effects of administered compounds on cardioplegia is required. In particular, an understanding of the changes in electrolytes through ion channels/transporters and the modulations of water channels would aid the understanding of heart damage. In the following section, we focus on the basic concept of electrolyte homeostasis as well as the changes of channel activity in cardiac tissue.

## 3. Altered Electrolyte Windows in the Cardiac Tissue

Na^+^-H^+^ exchangers (NHEs) are integral membrane glycoproteins that are expressed in most mammalian cells [39]. Members of the NHE family primarily regulate the intracellular pH but also regulate cell proliferation differentiation, apoptosis, migration, and volume control [40,41,42]. NHE contains 9 subfamilies (NHE-1 to NHE-9), which are composed of 600~900 amino acids [43]. In the heart, NHEs affect contractions by changing the intracellular pH. Furthermore, Na^+^ influx and H^+^ efflux through NHEs importantly restore the intracellular pH following ischemic acidification [2,44,45]. An Na^+^/H^+^ exchange transports Na^+^ in the forward direction under normal conditions, driven by an inwardly directed transmembrane Na^+^ gradient and, in exchange, expels a single proton; thus, it maintains the intracellular pH and regulates the intracellular Na^+^ levels [46]. However, during ischemia, an intracellular H^+^ accumulation provides a greater driving force. As a result, H^+^ leaks to the extracellular compartment and Na^+^ accumulates in the intracellular milieu. During ischemia, the transmembrane gradient was reduced by the accumulation of H^+^ in the extracellular compartment and this led to a decrease in the intracellular Na^+^ accumulation and NHE activity. During a reperfusion, the transmembrane gradient was restored by the removal of H^+^ in the extracellular compartment and H^+^ was excreted through the involvement of NHEs. This process arose from a sequential ion movement-like cascade reaction. Thus, the NHE transporters became reactivated during the reperfusion, which resulted in the intracellular Na^+^ accumulation [47] and stimulated the Ca^2+^ efflux through the Na^+^/Ca^2+^ exchanger (NCX). NCXs are plasma membrane-associated proteins that are driven by electrochemical gradients and allow Na^+^ to enter cells and Ca^2+^ to be released at a ratio of 3:1 [48]. Of the three NCX isoforms, NCX1 is expressed ubiquitously [49] whereas NCX2 and 3 are particularly expressed in the brain and skeletal muscles [50,51,52]. The three isoforms regulate Ca^2+^ homeostasis in a variety of cells and NCX1 is primarily involved with the heart [49,53]. Increased NCX1 levels in heart tissues are associated with human primary pulmonary hypertension and, when overexpressed, NCX1 decreases the SR Ca^2+^ load levels in heart failure [53]. Several studies have indicated that the NCX expression is associated with heart failure. NCX was overexpressed in heart failure animal models and a prolonged repolarization was revealed through monophasic action potentials (MAPs) in rodent chronic heart failure (CHF) [54,55]. Moreover, the overexpression of NCX prolonged the action potential duration [56] and this prolongation in heart failure sustained the time for the Ca^2+^ channel reopening, which may occur with a higher frequency of Ca^2+^ release from the SR [56,57]. In addition, the action potentials were shortened by inhibiting NCX with SEA0400 (a specific NCX1 inhibitor) [56]. In addition, inhibiting NCX shortened the MAP duration, reduced dispersion, and increased protection against the repolarization reserve and ventricular tachyarrhythmias (VTs) in rabbit heart failure [56]. In a forward mode, NCX releases Ca^2+^ whereas in a reverse mode, it enables Ca^2+^ to enter cells [58]. This reverse mode of NCX is induced by low intracellular Ca^2+^ levels and high intracellular Na^+^ levels in the failing heart [59,60]. Systolic and diastolic dysfunction and irreversible tissue injury induced by IR are associated with this intracellular Ca^2+^ accumulation [2,61,62]. Briefly, during ischemia, a reduced intracellular pH and ATP induced an excessive activity of NHE [63]. As a result, the intracellular Na^+^ was increased and cell swelling was induced whereas NCX was suppressed [61]. H^+^ was extruded during a reperfusion, resulting in a rapid increase in NHE activity and a reversed NCX was activated. The intracellular Ca^2+^ was increased again and an irreversible tissue injury occurred during the IR [63].

## 4. Bicarbonate Transporters and Their Associated Enzymes

In addition to cations such as H^+^ and Ca^2+^, the HCO_3_^−^ anion also plays an important role in heart tissues that express HCO_3_^−^ transporters such as the Cl^−^/HCO_3_^−^ exchanger SLC26A6, which is involved in HCO_3_^−^ influx and efflux [5,64]. SLC26A6 is a Cl^−^/HCO_3_^−^ exchanger and is mainly expressed in the kidneys and exocrine glands [65,66,67]. SLC26A6 is encoded by the *SLC26A6* gene and mediates the Cl^−^ influx and HCO_3_^−^ efflux. When SLC26A6 acts as an acid loader, it causes intracellular acidification by releasing HCO_3_^−^ from cells [68]. Mouse heart tissues predominantly express anion exchanger Slc26a6 [68]. In a previous study, we found the expression and activity of Slc26a6 were more increased in the cardiac tissues of db/db mice (a type 2 diabetes mellitus mouse model) than in normal cardiac tissues and that Slc26a6 enhancement induced an exacerbated intracellular acidification after a cardioplegia-induced arrest [5]. In another study, it was reported that Slc26a6-depleted cardiomyocytes exhibited sarcomere shortening and that the cardiac contractility was reduced [69].

HCO_3_^−^ is produced by the carbonic anhydrase (CA) catalyzed reaction between CO_2_ and H_2_O [70]. HCO_3_^−^ is a product of CA and a source of HCO_3_^−^ transporters. CAs are zinc metalloenzymes that hydrate carbon dioxide to bicarbonate and protons [71,72]. The CA family is composed of 5 types: α-, β-, γ-, δ-, and ζ-CAs. The β-ζ families of CA are non-mammal; therefore, we focused on α-CA. In mammals, it comprises CA I, CA II, CA III, CA VII, and CA VIII expressed in the cytosol; CA IV, CA IX, CA XII, and CA XIV expressed in the plasma membrane; CA VA and CA VB expressed in the mitochondria; and extracellularly secreted CA VI in the saliva and breast milk [73,74,75,76]. Although experimental evidence regarding cardiac CAs is sparse, CA II and CA IV were reportedly increased in hypertrophic ventricles and failing hearts in humans [77]. CA XIV interacted with the Cl^−^/HCO_3_^−^ exchanger in hearts and the CA XIV protein levels were enhanced in the hypertrophic hearts of rats [78]. In spontaneously hypertensive rats (SHR), CA XIV levels were reduced by benzolamide (a CA inhibitor), which reduced the HCO_3_^−^ flux to normal heart levels [78]. The roles of cardiac CAs require further study, especially regarding the modulation of the HCO_3_^−^ flux.

## 5. Understanding of Cardioplegia-Induced Water Transport

Cardiac water contents modulate the cardiac output [79] and a cardiac tissue edema is a likely risk factor of a reduced contractile force [80]. Furthermore, IR injury and inflammatory responses caused by CPB mediate myocardial edemas [81]. Thus, an understanding of the CPB-mediated water movement is required to properly address a cardiac injury. In an early study, elevated extracellular Ca^2+^ concentrations increased ischemic swelling in rat hearts and an inhibition of the Ca^2+^ channel with verapamil or bepridil reduced myocardial edemas [82]. Thus, an understanding of the water channels is needed in the heart field and how to regulate the water channels should be elucidated.

Aquaporins (AQPs) are traditionally integral plasma membrane proteins and water channels that move water or electrolytes through the cell membrane [83]. AQP-1 is expressed in mouse cardiac muscles, which also contain the mRNAs of AQP-3, -4, -5, -7, -9, -10, and -11 [79,84]. Briefly, AQP-3 transports water and glycerol and is expressed in most epithelial cells including those of the kidney, eye, brain, pancreas, respiratory tract, urinary tract, and the basal keratinocyte layer [85,86,87,88]. AQP-4 mediates the movement of water in the adult brain and neurons of the paraventricular and supraoptic nuclei [89] and is expressed in the kidneys, skeletal muscle, and stomach [90,91]. AQP-5 is present in the intercalated ducts and acinar cells of salivary glands [92,93,94] and is involved in salivary fluid secretion [95]. AQP-5 is expressed in sweet, lacrimal, and airway submucosal glands and in the lung and corneal epithelium [94,96,97]. AQP-7 is a 269-amino acid aquaglyceroporin and facilitates glycerol transport in adipose tissues [98,99]. AQP-9 is present in the liver and testis and facilitates the passage of water, glycerol, and urea [94,100]. AQP-7 and AQP-9 are associated with glycerol metabolism in adipose tissues and the liver [101]. AQP-10 is a 310-amino acid aquaglyceroporin expressed in the small intestine and transports water and glycerol [102,103]. AQP-11 is present in the proximal kidney and brain tubules [104,105] and plays an important role in ER-resident peroxiporin activity and transports glycerol and water through the ER membranes [106]. AQPs regulate the volume of cells in an osmolarity-dependent manner. Under hypotonic conditions, the cell volumes expand and subsequently rapidly decrease [107,108]. K^+^ efflux from cells is induced by K^+^ channel activation whereas water is released from the cells through AQPs by osmosis, resulting in a “regulatory volume decrease” [109], which protects the cells from rupture under hypotonic conditions. Under hypertonic conditions, the cells undergo shrinkage and then return to their original state, resulting in a “regulatory volume increase” [110]. The mechanism of cell shrinkage involves the activation of ion channels such as the Na^+^-H^+^ exchanger (NHE) and Na^+^-K^+^-Cl^−^ (NKCC), which allows Na^+^ to enter the cells [107] and the accompanying osmotic regulation induces water influx into the cells [111].

**Figure 2:**
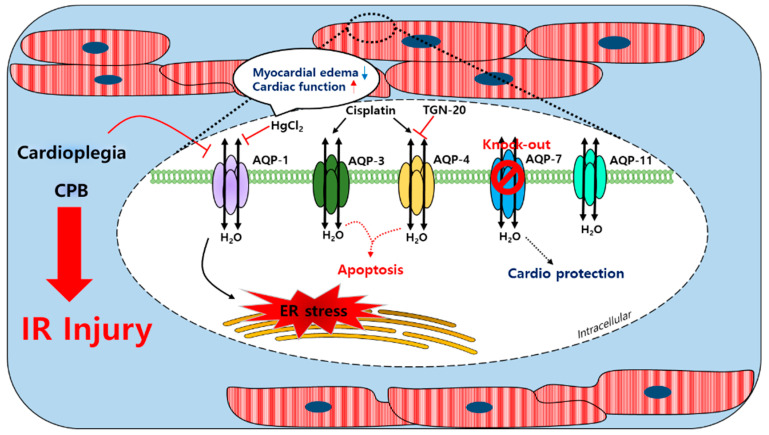
Schematic illustration of the involved myocardial aquaporin channel induced by cardioplegia and CPB. An IR injury induced by cardioplegia and CPB regulated the expressions of myocardial AQPs. The AQP-1 expression was increased by CPB and affected the ER stress; the expression could be regulated by HgCl_2_. Cisplatin enhanced the AQP-3 and AQP-4 expression and apoptosis was induced. TGN-20 suppressed the ischemia-induced AQP-4 expression. Cardioplegia decreased the AQP-1 expression but did not affect the expressions of AQP-4 or AQP-11. An AQP-7 knockout study revealed its cardioprotective effect. CPB: cardiopulmonary bypass; AQP-1/3/4/7/11: aquaporin-1/3/4/7/11; IR injury: ischemia/reperfusion injury; ER stress: endoplasmic reticulum stress; cisplatin: cis-dichlorodiammineplatinum.

AQP-1, AQP-4, AQP-7, and AQP-11 are found in the cardiac tissues of humans, rats, and mice; AQP-8 transcription was confirmed at the mRNA level in mice hearts [112,113,114,115,116]. Furthermore, AQP-1 expression in the sarcolemma was reported to be sensitive to ischemia-induced osmotic stress [79]. After a cardioplegic arrest in humans, cardioplegia induces a mild ischemic injury and reduces the mRNA levels of AQP-1 and glycosylation in the cardiac tissues [117]. AQP-4 expression was shown to be no different after ischemia in mouse hearts [79]. During CPB using an intermittent antegrade cold blood cardioplegia, the level of urine AQP-2 of the kidney collecting ducts was increased in patients with cardiac surgery [118]. Urine AQP-2 was associated with the arginine vasopression (AVP) level, which led to the activation of AQP-2 to promote the reabsorption of free water in the collecting duct. In HL-1 mouse cardiomyocyte cells, the expression of AQP-4 was increased under ischemia conditions and TGN-20, an AQP-4 inhibitor, decreased the mRNA and protein expression of AQP-4. In addition, the inhibition of AQP-4 reduced cardiomyocyte pyroptosis [119]. The treatment of cis-dichlorodiammineplatinum (Cisplatin), an anti-cancer drug, induced a myocardial edema as a side effect [120,121]. The expression of AQP-3 and AQP-4 was increased by cisplatin administration in the rat left ventricle and the expression of p-53 and BAX (apoptosis markers) was increased [122]. During CPB in an adult goat heart, an edema in heart tissues was associated with increases in AQP-1 mRNA and protein levels [123]; HgCl_2_ was suppressed by HgCl_2_, which did not affect the expression of AQP-1 and improved the cardiac function. Thus, the myocardial water content was more increased in the goat heart CPB groups than in the control groups [123]. The decrease of the intracellular pH of the myocardial tissue and plasma in patients with a severe aortic stenosis after CPB was associated with ischemia and revealed that the expression of AQP-1 and AQP-4 was increased and a myocardial edema was induced [124]. More recently, the coordination between AQP-1 and the tight junction protein connexin 43 was associated with the pathological development of a myocardial edema [123]. The regulation of the cellular volume was achieved using the water channels and several related transporters [125]. AQP-7 transports water and small molecules such as glycerol. During hyperkalemic cardioplegia using St. Thomas’ Hospital solution No. 2 (STH2) in aquaporin-7 KO mice, the left ventricle advanced pressure was found to be lower than in WT mice and the Troponin T levels were significantly reduced in isolated hearts [126], which suggested that an AQP-7 deficiency protected the myocardium and reduced the risks of complications caused by cardiac surgery during hyperkalemic cardioplegia. A schematic of the actions of the AQP channels in the cardiac tissue is provided in Figure 2. Currently, it is unclear whether the cardiac water channels work alone or with other transporters during CPB. Conceivably, further studies are required on the coordination between the water channels and the ion transporters in the context of a cardiac injury.

## 6. Conclusions and Future Works

Cardioplegia is used to maintain a normal cardiac function during cardiac surgery; however, the procedure has various side effects. As shown in Table 1, despite the efforts made, myocardial protection during a cardioplegia-induced arrest remains a challenging issue. Previously, we reported that an ion imbalance occurred in cardioplegia-exposed cardiac tissue and that this was more severe in diabetic hearts [5]. Furthermore, it appears that modulations of the electrolyte movements through ion channels such as SLC26A6 and its related enzyme, carbonic anhydrase, as well as through ion transporters such as NCX, NHE, and AQPs, play an important role in cardiac physiology. In our previous study in other tissues, the joint edema showed in rheumatoid arthritis (RA) patients was caused by the osmotic change of the synovium, where it was addressed that not only AQP-1 but also a combined ion transporter such as Na^+^-K^+^-2Cl^−^ co-transporter 1 (NKCC1) and osmotic regulatory protein oxidative stress-responsive kinase 1 were involved [127,128]. A cytotoxic edema was induced by a pro-inflammatory cytokine such as IL-6 in RA synovial fluids from RA patients and NKCC1 was recruited to the plasma membrane of RA fibroblast-like synoviocytes and a combined volume regulation through AQP-1 was mediated [128]. In this regard, we considered the sufficient possibility that a cardiac edema caused by an IR injury after a cardiac arrest is associated with the convergent involvement of dynamic ion transporters and AQP. Conceivably, the severity of the IR injury might be enhanced by the involvement of dysregulated convergent channels/transporters. Although the field of cardiac channelopathy is relatively new and ion regulation through channels and transporters is complicated, we hope that this review provides a few unexpected clues and a medicinal approach. We believe that the delicate tuning of an ion imbalance might resolve the myocardial dysfunctions induced by cardioplegia and membrane-associated cardiac channels and transporters will provide favorable advanced strategies to achieve a clinical use in the cardiac field.

## Figures and Tables

**Table 1:** Characteristics of the modified cardioplegia solutions.

Drugs	Solutions	Limits	Mechanisms	Ref.
Adenosine triphosphate-sensitive K^+^ channel (K_ATP_) opener diazoxide	Hyperkalemic cardioplegia	Myocyte swelling and reduced contractility	Diazoxide prevented myocyte swelling and reduced contractility by blocking the K_ATP_ channel related to myocardial stunning	[33]
Cyclosporine A (MPTP-opening inhibitor)	Cold antegrade crystalloid cardioplegia	Apoptosis-related alteration in the mitochondrial structure	Promoted Bax translocation and inhibited calcineurin related to Ca^2+^ homeostasis	[16]
AMPK activator (AICAR, metformin)	Deoxygenated hypothermic cardioplegia	Cardiomyocytic apoptosis	Enhanced anti-apoptotic proteins (Grp78) and decreased ER stress	[19]
Pyruvate-enriched cardioplegia	Crystalloid cardioplegia	Inflammation that can damage the myocardium	Attenuated oxidative stress during CPB and increased TIMP-2	[35]
L-arginine cardioplegia	Cold blood + anterograde and retrograde cardioplegia	Myocardial ischemic damage	Production of nitric oxide, increased IL-2 receptor, IL-6, and tumor necrosis factor levels	[31]
Bradykinin	Cold crystalloid cardioplegia	Apoptosis under cardiopulmonary bypass	Decreased NO level and nuclear translocation of NF-κB	[19]
Dilong (earthworm)	High KCl cardiologic solution	Apoptosis of cardiomyoblast (H9c2 cells)	Attenuated caspase-3 activation and enhanced PI3K/Akt and Bcl-2	[38]
Urocortin	Cold blood cardioplegia	Apoptosis and dysfunction in diabetic hearts	Induction and mitochondrial relocation of PKC-δ	[27]
NHE-1 inhibitor eniporide	Cold cardioplegia	Ca^2+^ overloading	Inhibition of NHE-1 reduced the infarct size after hypothermic ischemia	[18]

K_ATP_: adenosine triphosphate-sensitive potassium channel; MPTP: mitochondrial permeability transition pore; Bax: Bcl-2-associated X; AMPK: AMP-activated protein kinase; AICAR: 5-aminoimidazole-4-carboxamide ribonucleoside; Grp78: glucose-regulated protein; ER: endoplasmic reticulum; CPB: cardiopulmonary bypass; TIMP-2: tissue inhibitor of metalloproteinase 2; IL-2 receptor: interleukin-2 receptor; IL-6: interleukin-6; NO: nitric oxide; NF-κB: nuclear factor kappa-light-chain-enhancer of activated B cells; PI3K/Akt: phosphoinositide-3-kinase/protein kinase B; PKC-δ: protein kinase C-δ; NHE-1: Na^+^-H^+^ exchanger-1.
